# Cell migration, DNA fragmentation and antibacterial properties of novel silver doped calcium polyphosphate nanoparticles

**DOI:** 10.1038/s41598-023-50849-z

**Published:** 2024-01-04

**Authors:** Basma Ekram, Emad Tolba, Ahmed F. El-Sayed, Werner E. G. Müller, Heinz C. Schröder, Xiaohong Wang, Bothaina M. Abdel-Hady

**Affiliations:** 1 Polymers and Pigments Department, Chemical Industries Research Institute, National Research Centre, Cairo, 12622 Egypt; 2 Microbial Genetics Department, Biotechnology Research Institute, National Research Centre, Cairo, 12622 Egypt; 3 Egypt Center for Research and Regenerative Medicine (ECRRM), Cairo, Egypt; 4 ERC Advanced Investigator Grant Research Group at the Institute for Physiological Chemistry, University Medical Center of the Johannes Gutenberg University, Duesbergweg 6, 55128 Mainz, Germany

**Keywords:** Materials science, Nanoscience and technology, Biomaterials

## Abstract

To combat infections, silver was used extensively in biomedical field but there was a need for a capping agent to eliminate its cytotoxic effects. In this study, polymeric calcium polyphosphate was doped by silver with three concentrations 1, 3 or 5 mol.% and were characterized by TEM, XRD, FTIR, TGA. Moreover, cytotoxicity, antibacterial, cell migration and DNA fragmentation assays were done to assure its safety. The results showed that the increase in silver percentage caused an increase in particle size. XRD showed the silver peaks, which indicated that it is present in its metallic form. The TGA showed that thermal stability was increased by increasing silver content. The antibacterial tests showed that the prepared nanoparticles have an antibacterial activity against tested pathogens. In addition, the cytotoxicity results showed that the samples exhibited non-cytotoxic behavior even with the highest doping concentration (5% Ag-CaPp). The cell migration assay showed that the increase in the silver concentration enhances cell migration up to 3% Ag-CaPp. The DNA fragmentation test revealed that all the prepared nanoparticles caused no fragmentation. From the results we can deduce that 3% Ag-CaPp was the optimum silver doped calcium polyphosphate concentration that could be used safely for medical applications.

## Introduction

Bacterial tissue infections can be a significant challenge to successful in wound healing, bone fracture repair and other orthopedic-related surgeries associated with a prosthetic or osteosynthetic device that used to repair damaged bone tissue or arrest the spread of diseases^1,2^. The available treatment options such as autografts, allografts, and synthetic materials are based on replacing of the injured bone tissue via bone-like materials of natural or synthetic origin, followed by the systemic administration of antibiotics to reduce bacterial growth and chronic implant-related bone^3–5^. However, their effectiveness can vary depending on the infection situation, such as the loss of tissue-migrating capacity of antibiotics due to granulation or biofilm formation on the implant's surface due to bacterial infections which inhibits their functionality^6,7^. Nowadays, the combination of antimicrobial agents within bone replacement material for localized delivery of antibiotics offers considerable advantages over these traditional methods^8–10^. However, many of them lack sustained release for prolonged periods that prevents proper bone formation and also they are not effective to all the present types of bacteria^11^. Therefore, alternative and innovative treatment approaches are clinically recommended via engineering multifunctional materials which are able to fight against bacterial infections and integrate with the host tissue, whilst exhibiting controlled resorption rate with an ultimate replacement by regenerating tissue^12,13^.

Silver nanoparticles (AgNPs) have attracted a great attention in the last decade for its broad antimicrobial activity against both gram-positive and gram-negative bacteria, viruses, fungi and even antibiotic-resistant bacterial strains^14,15^. Hence, physicians used silver compound and colloids as a strong and effective antibacterial agent for the treatment of ophthalmic problems, burns, ulcerations, and infected wounds^16–18^. Today, many silver formulations exist in the market in different formulation, including colloidal solution, topical creams and gels to control bacterial infections for better effective treatment effects^19–22^. Even though, AgNPs exhibit nontoxicity effect towards different human cells at low concentrations^23,24^. Recent in vitro and in vivo study outcomes suggest that the significant risks exerted by metal nanoparticles, in particular AgNPs, is mainly based on cellular oxidative stress due to the induction of the Reactive Oxygen Species (ROS) and that the physiochemical properties of NPs (size and shape) as well as cumulative release profile of NPs are known to modulate their dynamic interaction with plasma proteins and cells and their organelles and, possibly, their cytotoxicity^25,26^. Engineered material-containing NPs may represent an effective approach that could be used to control release of Ag ions without significant harmful to mammalian cells^27,28^. As a result, researchers investigated silver doping as an alternative less cytotoxic way of incorporating silver^27,29–31^.

As a previous study by Peetsch et al., they synthesized spherical silver-doped calcium phosphate nanoparticles using a co-precipitation process and they found that the toxic silver concentrations were found to be in the range of 1–3 μg mL^−1^ for all tested cells and strains^31^. Additionally, a study by Singh and Batra prepared 3% silver-doped calcium phosphate nanopowder and it showed antimicrobial activity^30^. Another study by Sugiura et al., they synthesized silver-substituted octacalcium phosphate powder that showed antibacterial properties with low cytotoxicity at the optimized concentration of 1% Ag^32^. Nie et al. also prepared silver doped biphasic calcium phosphate by adding silver nanoparticles to an emulsion of alginate and biphasic calcium phosphate. The produced microclusters showed cytocompatibility besides its antibacterial properties^33^.

Polyphosphates (polyP), a unique type of inorganic biodegradable polymer, which are linear chains of orthophosphate molecules, have primarily been studied in the context of prokaryotic organisms^34^. Some studies have suggested a role for polyphosphates in bone development, as they are found in higher concentrations in cells that form bone than in those that form soft tissue^35^. Additionally, researchers have shown that the addition of exogenous polyphosphates to certain cell cultures can promote calcification and increased expression of certain genes related to bone formation^34^.

Recently, calcium polyphosphate (CaPp) has drawn attention as a type of polyphosphates due to its similar chemical elements to bones, the multiple physiological function of polyP and a source for calcium and phosphate ions^36,37^. It has higher osteoinductive activity compared to calcium phosphate^36^. Unlike, calcium phosphate, CaPp contains polyP which serves as a source of metabolic energy so it can stimulate the development of bone cells and activates the alkaline phosphatase gene, which is an indicator of osteoblast formation besides its regulation to ATP levels in bone cells^38–40^.

However, there have been previous efforts to synthesize silver-doped calcium phosphate particles as previously mentioned, but to the best of our knowledge, no work has demonstrated the doping/embedding of silver in amorphous calcium polyphosphate, in addition neither its effect on DNA fragmentation nor wound healing study were studied before^28–31^.

In this work, silver nanoparticle-loaded amorphous calcium polyphosphate NPs for biomedical applications were prepared via wet chemical precipitation as it is one of the most feasible ways to produce homogenous nanoparticles^41^. Consequently, the physicochemical properties of the prepared silver doped CapolyP NPs were characterized by electron microscopes (SEM and TEM) and FTIR and in vitro cell toxicity and migration assays were tested in vitro against fibroblast cells.

## Material and methods

### In-situ precipitation of calcium polyP doped silver nanoparticles (Ag-CapolyP NPs)

Calcium polyphosphate doped silver nanoparticles of a [Ca + Ag]/P molar ratio of 1 were synthesized via wet chemical precipitation reaction between aqueous solution of calcium nitrate tetrahydrate (Ca(NO_3_)_2_.4H_2_O, 99.9%, Sigma Aldrich), sodium polyphosphate (NapolyP; (NaPO_3_)_40_; Chemische Fabrik Budenheim) and silver nitrate (AgNO_3_, Sigma Aldrich) as mentioned in our previous study with some modifications^38^. In brief, one gram of NapolyP were dissolved in distilled water and kept for 30 min under stirring at pH 10 using NaOH solution (1 M) to prevent hydrolysis of the polyP. In parallel, calcium nitrate (10 mmol) was suspended in 50 ml distilled water into which 0, 0.1, 0.3 or 0.5 mmol silver nitrate (corresponding to 0, 1, 3 or 5 mol.% respectively) was added and stirred at 500 rpm at pH 10 for 30 min. Afterwards, the [Ca + Ag]-containing solutions were added drop by drop to the polyP containing solutions. The pH value was constantly adjusted at 10 during the reaction. The reaction was all done at room temperature until the [Ca + Ag]-containing solutions were completely dropped into the polyphosphate solution. The obtained mixtures were stirred for further 6 h away from light to prevent oxidation of silver ions. Finally, the precipitated nanoparticles were collected using centrifuge (6000 rpm) and cross washed with ethanol and water 4 times to remove unreacted reagents and then dried at 90 °C within a vacuum oven. The obtained Ag-CaPp NPs gel were stored at 4 °C till further using.

### Microstructure characterizations

The samples were tested by X-ray diffractometer (XRD) (MiniFlex-600, Rigaku Corporation, Japan) with scanning angle from 5–80° at scanning speed of 10°/min. Fourier-transform infrared (FTIR) spectroscopy was performed in the wavenumber range of 4000–500 cm^−1^ with an attenuated total reflectance-FTIR spectroscope/Varian IR spectrometer (Agilent, Santa Clara; CA). Thermal analysis (TGA) of the sample was performed using an SDTQ600 analyzer with a rate of 10 °C min^−1^ under argon in temperature range from 50 to 500 °C.

### Antimicrobial screening

Antibacterial activity was tested according to Magaldi et al. by agar well diffusion method^42^. Three different bacterial strains were used as test antipathogenic activity which are *Staphylococcus aureus, Enterococcus faecalis and E. coli*. The obtained pure cultures of these pathogens were subcultured in nutrient broth and incubated at 37 °C on a rotary shaker at 120 rpm for 24 h. After washing the subcultured strains with 0.9% saline solution until the intensity of strain was obtained at 0.5 optical density at 570 nm. Then each strain was swabbed uniformly on the individual Mueller–Hinton agar plates using sterile cotton swabs and a well was made on agar plates using gel puncture. Then the 100 μl of different chemical compounds samples were added into the respective well. The zone of inhibition was measured using a zone scale after 24 h incubation at 37 °C.

### Cell viability assay

Human Skin Fibroblast (HSF) was purchased from Nawah Scientific Inc., (Mokatam, Cairo, Egypt). HSF cells were cultured in DMEM media supplemented with 100 mg/mL of streptomycin, 100 units/mL of penicillin and 10% of heat-inactivated fetal bovine serum in humidified, 5% (v/v) CO_2_ atmosphere at 37 °C. The cells were harvested from passage 5 at nearly 70% confluency. Cell viability was assessed by Sulforhodamine B (SRB) assay^43^. Aliquots of 100 μL cell suspension (5 × 10^3^ cells) were in 96-wellplates and incubated for 24 h. Cells were treated with another aliquot of 100 μL media containing Ag-CapolyP NPs samples at various concentrations. After 72 h, cells were fixed by replacing media with 150 μL of 10%TCA and incubated at 4 °C for 1 h. The TCA solution was removed, and the cells were washed 5 times with PBS. Aliquots of 70 μL SRB solution (0.4%w/v) were added and incubated in a dark place at room temperature for 10 min. Plates were washed 3 times with 1% acetic acid and allowed to air-dry overnight. Then, 150 μL of TRIS (10 mM) was added to dissolve protein-bound SRB stain; the absorbance was measured at 540 nm using a BMGLABTECH^®^-FLUOstar Omegamicroplate reader (Ortenberg, Germany).

### Scratch assay

HSF cells were plated at density 3 × 10^5^/well onto a coated 6-well plate and cultured overnight in 5% FBS-DMEM at 37 °C and 5% CO_2_. On the next day, horizontal scratches about 3 mm were introduced into the confluent monolayer; the plate was washed thoroughly with PBS, control wells were replenished with fresh medium while the nanoparticles containing wells were treated with fresh media containing nanoparticles. Images were taken using an inverted microscope at the indicated time intervals (0, 24, 48, 72 h). The plate was incubated at 37 °C and 5% CO_2_ in-between time points. The acquired images are displayed below and were analyzed by MII Image View software version 3.7 to measure the changes in the scratch width. Migration rate is calculated by dividing the time spent in migration according to the formula below:

$${\text{Migration rate R}}_{{\text{m}}} \, = \,{\text{W}}_{{\text{i}}} \, - \,{\text{W}}_{{\text{f}}} /{\text{t}},$$

where Rm is the rate of cell migration, Wi is the average initial wound width, Wf is the average final wound width, and t is duration of migration (in hours).

### DNA fragmentation

The DNA fragmentation assay is a widely used technique to measure apoptosis or programmed cell death^44^. In this study, the test was performed to study the effect of the prepared nanoparticles to DNA fragmentation (damage) to ensure its safety. Pheochromocytoma cells (PC12) are commonly used for this assay because they demonstrate apoptotic characteristics in response to various stimulations^45,46^. This makes them an ideal model system to study the mechanism of apoptosis and how it affects DNA integrity. Their responsiveness to various stimuli and ability to differentiate make them an ideal model system for studying the complex interplay between apoptosis and DNA damage^47^. PC12 cells were grown at 37 °C (5% CO_2_ atmosphere) in RPMI 1640 medium supplemented with 10% heat-inactivated horse serum, 5% heat-inactivated fetal bovine serum, 2 mM glutamine, 1 mM sodium pyruvate, 100 U/ml of penicillin and 100 mg/ml of streptomycin. DNA fragmentation was measured after extraction of low molecular weight DNA. Briefly, PC12 cells at a density of 2 × 10^6^ cells/well in 6-well plates were incubated with different chemical compounds for 48 h at 37 °C in 5% CO_2_ and 95% air. 2 X 10^6^ were resuspended in 500 μL Tris–EDTA bufferand lysed with 0.2% Triton X-100. DNA was precipitated in ethanol for 6 h in the presence of 0.5 M NaCl. The high molecular weight fraction was sedimented by high-speed centrifugation, and the fragmented DNA was extracted from the aqueous phase with phenol and chloroform and then precipitated with isopropanol. After resuspension in Tris–EDTA buffer, the samples were incubated with RNAse A (0.1 mg/ml) for 30 min at 37 °C. Finally, DNA was electrophoresed using 1% agarose gel and visualized by ultraviolet light. This method can be used to semi-quantify the degree of DNA fragmentation, when started with the same cell number. DNA electrophoresis was carried out at 15 V for 2 h, and DNA was stained with ethidium bromide (EtBr; Sigma-Aldrich Corp.) and then examined and photographed under UV light box.

## Results

### Morophological analysis

**Figure 1:**
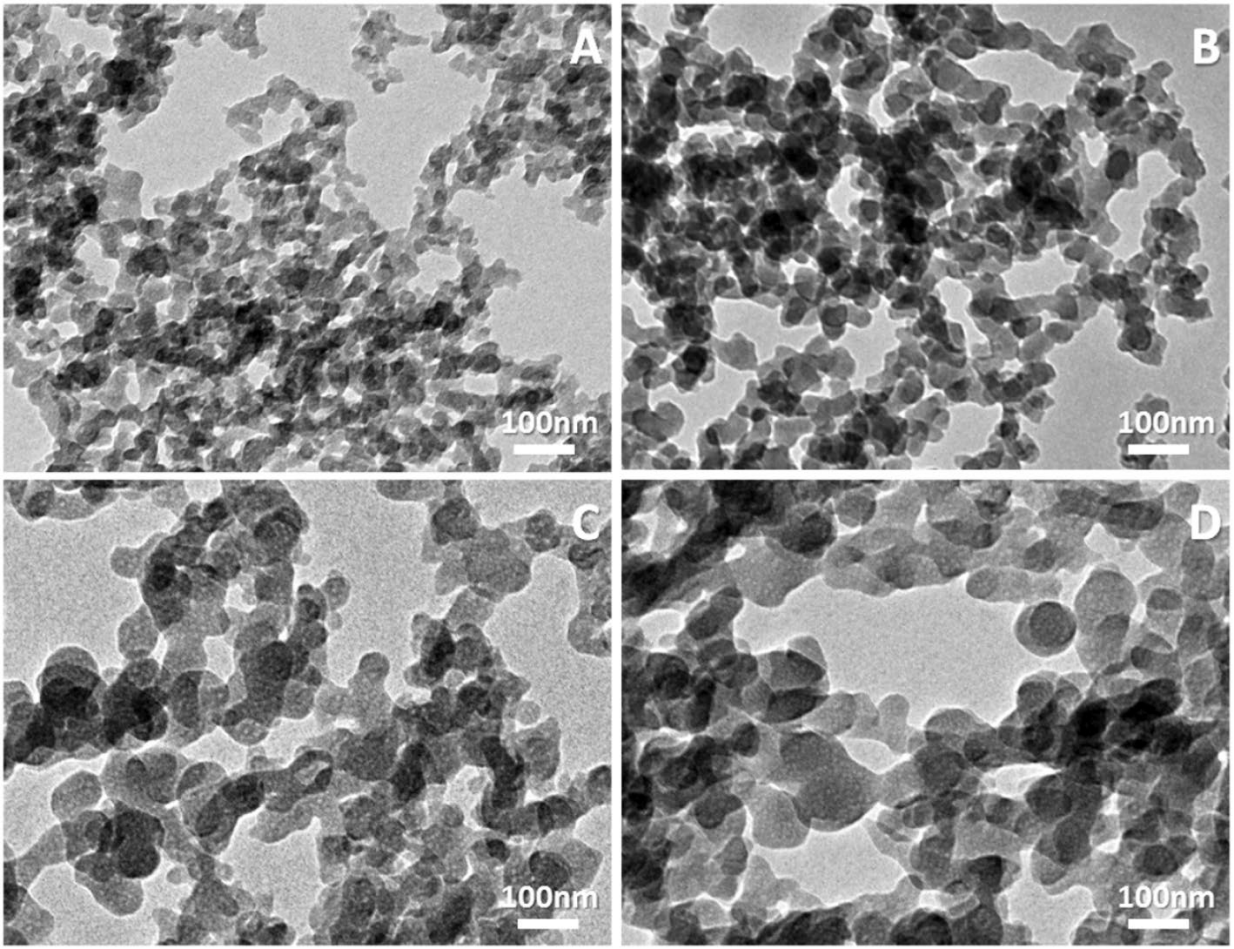
TEM images of the synthesized nanoparticles (**A**) CaPp, (**B**) 1.5% Ag- CAPp, (**C**) 3% Ag-CaPp and (**D**) 5% Ag-CaPp.

Transmission Electron Microscopy (TEM) images were captured to study the effect of different percentages of silver doping on the particle size of the nano calcium phosphate. Particle size affects the cellular intake of the nanoparticles and hence its effect^48^. The TEM images of the prepared nanoparticles are shown in Fig. 1. In general, the obtained particles exhibited high aggregation tendency and near-spherical shape and are all in the nano-range. Aggregation may be due to the surface charge of the prepared nanoparticles and it can be overcame by excessive sonication^49^. Aggregation of the nanoparticles may hinder the nanoparticles cellular uptake^50^. However, high cellular uptake of silver is not advantageous, so it is important to maintain the uptake to a certain limit to get its advantages and keep out its drawbacks^51^. Furthermore, the mean diameter for CaPp is 28 ± 8 nm, however the increase of silver ions concentration in the precursor solutions results in an increase of the mean particle diameter to 53 ± 8 nm for 1.5%Ag-CaPp, 71 ± 14 nm for 3%Ag-CaPp and 100 ± 21 nm for 5%Ag-CaPp samples. The increase in particle size may be attributed to the difference in ionic radius between calcium and silver (0.128 nm for silver vs 0.099 nm for calcium) as the larger ionic, the larger particle size^52,53^. Even though XRD patterns detects silver in metallic, rather than ionic form in the synthesized powder, no silver particles were visible in the TEM images, which provides some support for silver particles being very small and well dispersed within the CaPp particles.

### Structural analysis

**Figure 2:**
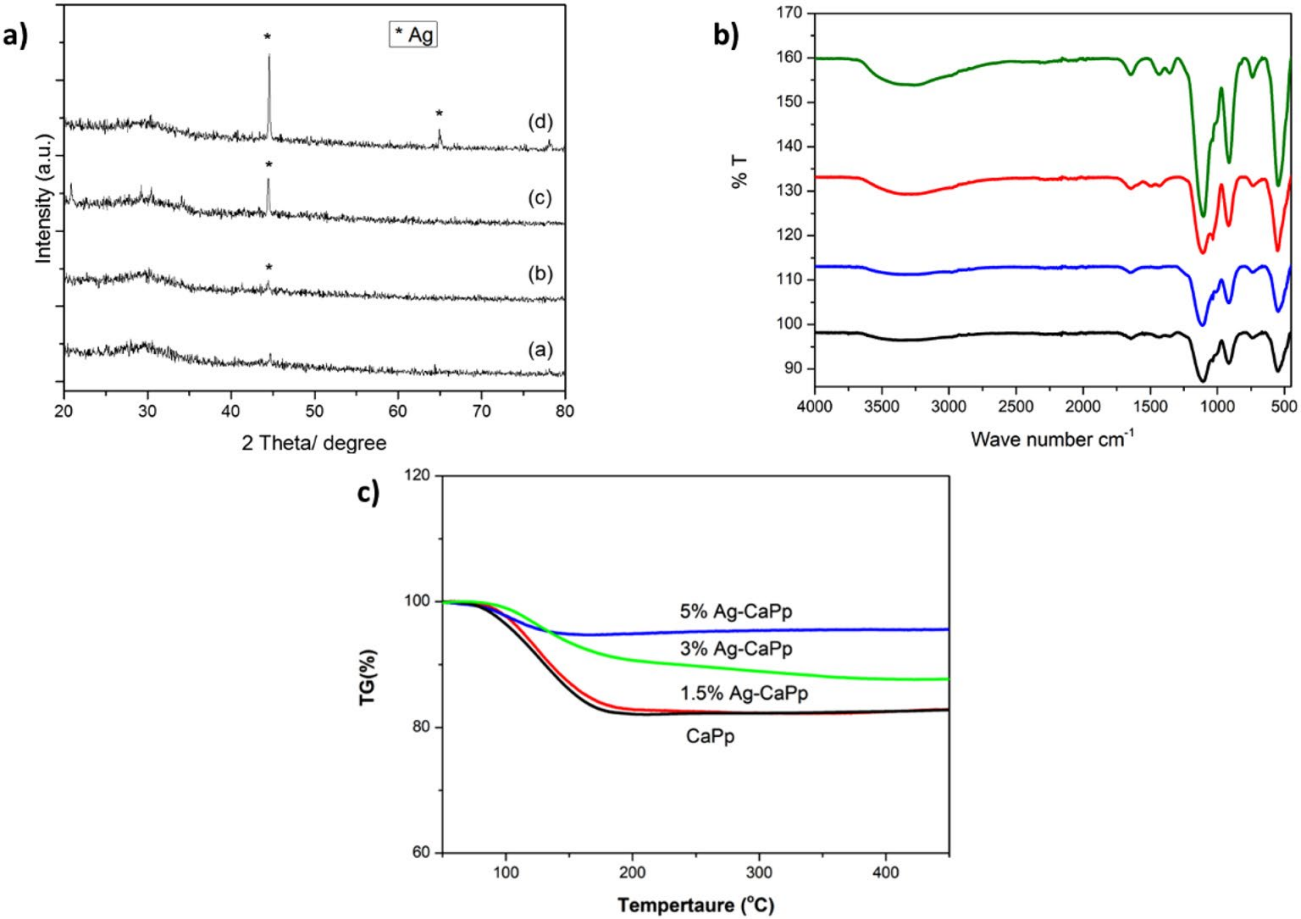
(**a**) XRD patterns of as-prepared (**a**) CaPp, (**b**) 1.5% Ag-CAPp, (**c**) 3% Ag-CaPp and (**d**) 5% Ag-CaPp nanoparticles, (**b**) The FTIR Spectra of all the prepared CaPp and AgCaPp nanoparticles and (**c**) The TGA curve of all the prepared nanoparticles.

Structural analysis was performed to identify the crystal structure, the change in functional groups with the possible interactions and also the thermal stability of the prepared nanoparticles to ensure the formation of doped silver-calcium polyphosphate. X-ray diffraction was examined to test the crystal nature of the samples. The X-ray diffraction pattern of pure CaPp and the Ag-CaPp NPs of different silver content is shown in Fig. 2a. The XRD pattern of CaPp and 1.5 Ag-CaPp samples showed no crystalline phases which reveal their amorphous nature. By increasing silver content, the XRD patterns of 3%Ag-CaPp and 5%Ag-CaPp samples showed some distinguishing peaks at 2θ = 44.77° 64.77° and 77.82° corresponding to metallic silver nanoparticles NP (JCPDS # 087–0719) which prove the incorporation of silver in the prepared nanoparticles.

The FTIR spectra are shown in Fig. 2b. The peaks at 3000 and 1637 cm^−1^ are attributed to the OH stretching and bending vibrations of absorbed water, respectively. The band around 1120 cm^−1^ assigned to the PO_2_ asymmetric stretching. The asymmetric and symmetric stretching of PO_3_ found at chain ends were noticed as very small peaks at 1030 and 1015 cm^−1^ are assigned to the asymmetric and symmetric stretching modes of chain-terminating (PO_3_) groups. The band at 902 cm^−1^ is attributed to the asymmetric stretching modes of the P–O–P linkages. In addition, at the peak at 557 cm^−1^ is characteristic bending PO_4_^54^. There was a noticed increase in the phosphate bands by the increase in the silver content, this may be due to the ionic crosslinking of silver between non bridging oxygen of the polyP phosphatate groups causing increased interaction between the PolyP chains^55^.

Thermogravimetric analysis (TGA) curve of pure CaPp and the 3 different concentrations (1.5, 3, 5% Ag-CaPp) are shown in Fig. 2c. At the TGA curve, five different stages were observed during the total weight loss. The first stage of weight loss is in between room temperature up to 150 °C, which is due to the loss of physically adsorbed water. The second stage of weight loss is from 150 to 450 °C and this is due to loss of chemisorbed water. From the curve it was shown that by the increase in silver percentage, there was an increase in thermal stability. The results agree with results obtained by Singh and Batra in their study on the effect of doping of calcium phosphate with silver^30^.

### Antimicrobial activity

To overcome the antibiotics resistant bacteria, an alternative drug invention is necessary. Usage of some metals as a source of antibacterial activity could be one of the alternatives options against various microbial contaminations. In particular, silver is effective against a broad range of bacteria due to its ability to damage their cell walls and membranes, leading to their death^29,30^. Silver can be released from silver-containing compounds, including silver nanoparticles, which are becoming increasingly popular due to their unique properties.

**Figure 3:**
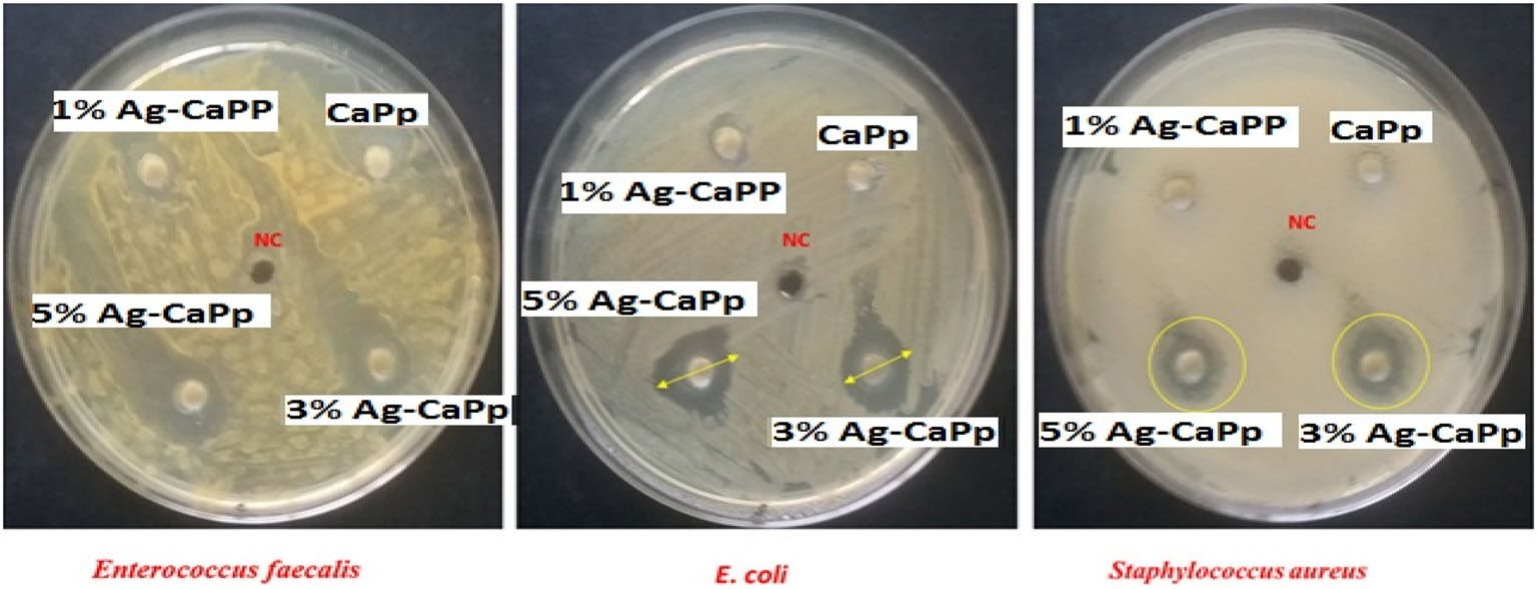
Antimicrobial activity of the prepared nanoparticles by well diffusion method against *E. coli, Staphylococcus aureus,* and* Enterococcus faecium.*

In our study, the silver doped CaPp showed more excellent antimicrobial activity against all tested microorganisms as shown in Fig. 3, and Table 1. The 3% and 5% Ag-CaPp samples produced a potent inhibition in the respective well at the zone of inhibition which were 12.8 ± 0.7 and 14.5 ± 0.9 mm for Enterococcus faecalis, respectively. While they produced moderate inhibition which were 6.3 ± 0.2 and 8.6 mm for E. coli respectively but the least inhibition was 3.7 ± 0.1 and 4.3 ± 0.3 for *Staphylococcus aureus*, respectively. Also, our results indicated that CaPp has not any activity against all tested pathogen microorganisms, however 1.5% Ag-CaPp showed slight antibacterial effect about 2.3 ± 0.1 mm against E. coli and 1.3 ± 0.1 mm against *Staphylococcus aureus*. To examine the effect of different samples on antimicrobial activity, we conducted a two-way ANOVA. Our results showed that the different samples and the selected strains significantly affected antimicrobial activity, with a high effect size. The p-values were less than 0.005 for the different samples and pathogenic microorganisms. Furthermore, the interaction between the two independent factors also significantly affected antimicrobial activity with a p-value of less than 0.005. Overall, the mean difference of inhibition zones between different samples was found to be statistically significant against different pathogenic microorganisms. As indicated, the increase in silver doping percentage showed higher antimicrobial effect due to the antibacterial properties of the silver^30^. Hence, based on our results both sample 3 and 5% Ag-CaPp possesses effective antimicrobial activities against the studied pathogens so they can be used in as an antibacterial biomaterial that can fight different types of drug resistive bacteria with a prolonged effect.

**Table 1:** Antimicrobial activity of the samples evaluated by well diffusion method.

No	Microorganisms	Type	Antimicrobial activity (mm)				
			Control [DMSO]	CaPp	1.5% Ag-CaPp	3% Ag-CaPp	5% Ag-CaPp
1	*Staphylococcus aureus*	G + ve	(−)	(−)	( ) 1.3 ± 0.1	(+) 3.7 ± 0.1	(+) 4.3 ± 0.3
2	*Enterococcus faecalis*	G + ve	(−)	(−)	( ) 3.6 ± 0.3	(+) 12.8 ± 0.7	(+) 14.5 ± 0.9
3	*E. coli*	G − ve	(−)	(−)	( ) 2.3 ± 0.1	(+) 6.3 ± 0.2	(+) 8.6 ± 0.5

(G + ve) Gram-Positive, (G − ve) Gram-Negative, (+) Positive, (−) Negative.

### Cell viability and scratch assays

**Figure 4:**
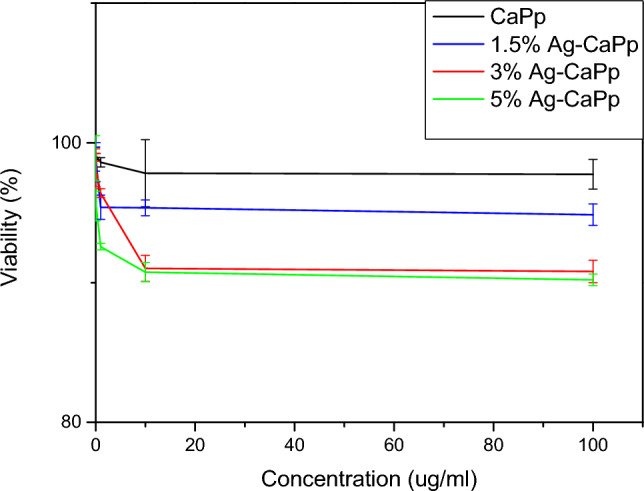
Cell viability of the different prepared nanoparticles at their different concentrations.

Cell viability was determined by SRB assay and reported at Fig. 4. The cytotoxicity values at the highest nanoparticles concentration 100 µg/ml is found to be 97.75 ± 1.06, 94.86 ± 0.77, 90.8 ± 0.8 and 90.2 ± 0.41 for CaPp, 1.5% Ag-CAPp, 3% Ag-CaPp and 5% Ag-CaPp nanoparticles respectively. To examine statistical significance, one-way Anova test was performed for the different results of the samples at 100 µg/ml as it is the highest concentration. All the results are statistically significant to each other (p-value < 0.0001) except for 3% and 5% Ag-CaPp, there are no statistical difference between them (p-value > 0.05). According to a cytotoxicity study, all the samples showed non cytotoxic (viability above 90%) behavior even with the highest doping concentration (5% Ag-CaPp) which indicated its biocompatibility and safety of the prepared nanoparticles to be used in different medical applications such as bone grafting and wound healing applications.

**Figure 5:**
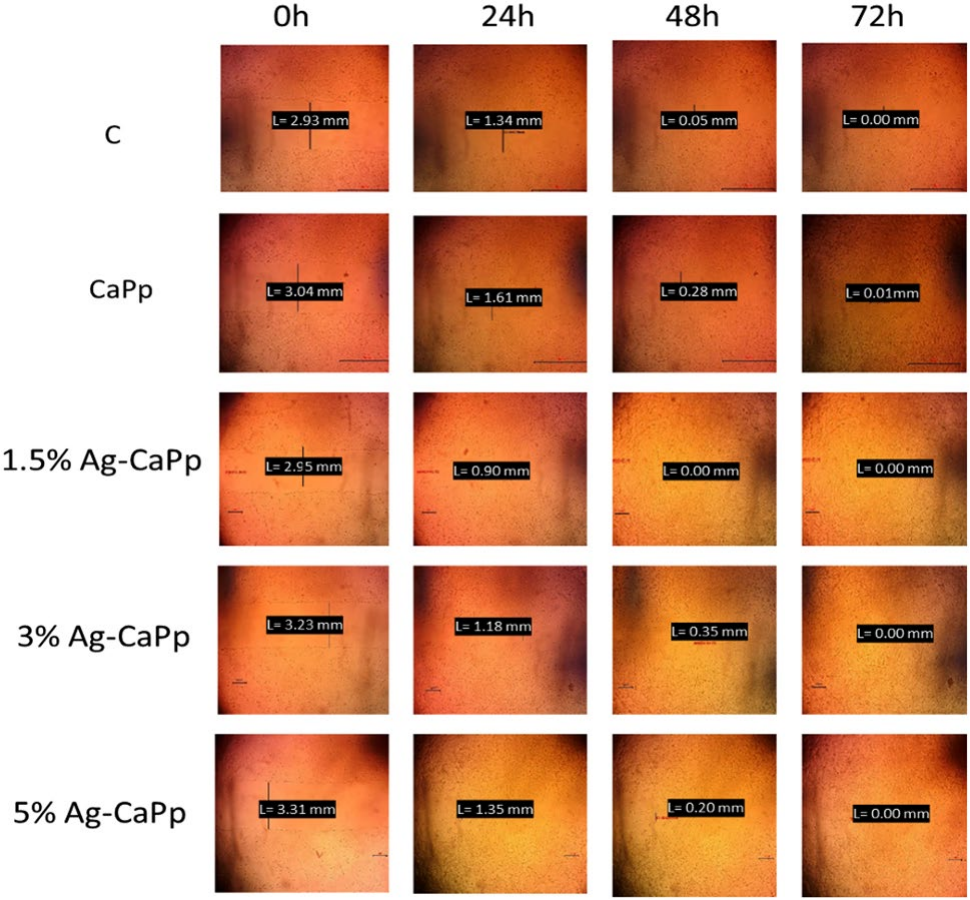
Light microscope images cell migration after 0, 24, 48 and 72 h for all samples.

**Figure 6:**
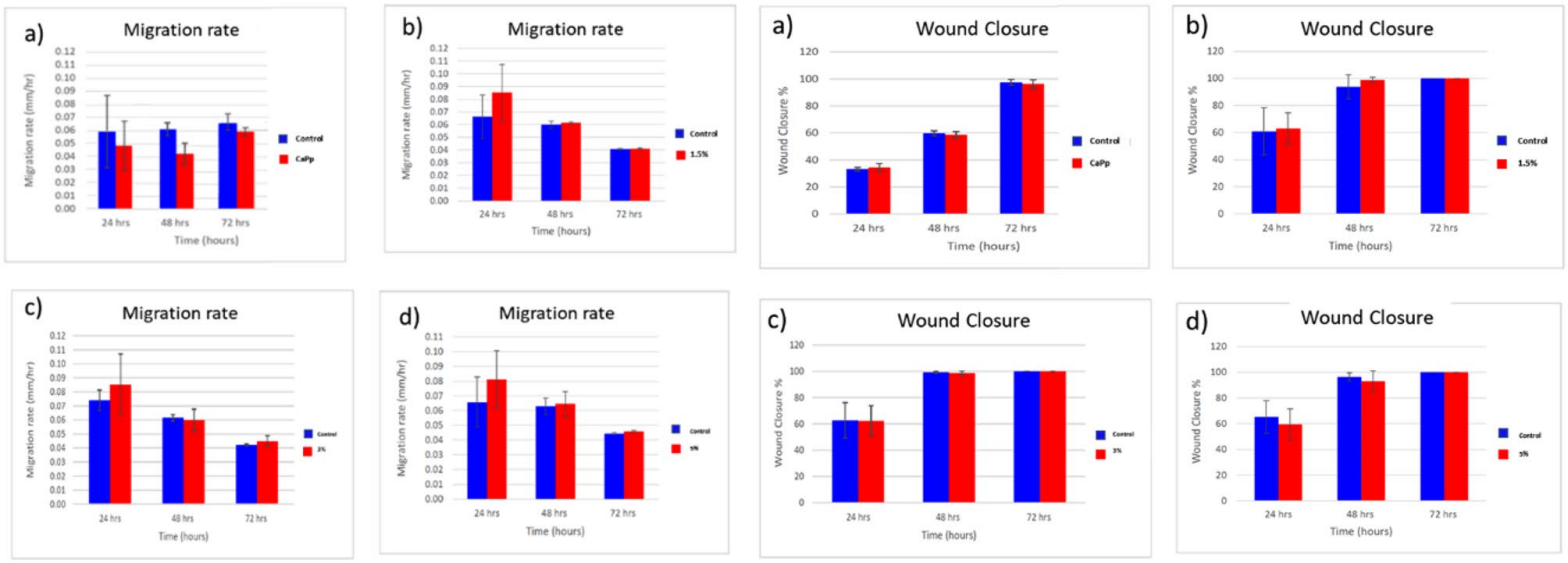
Migration rate and wound closure percentage of (**a**) CaPp, (**b**) 1.5% Ag-CAPp, (**c**) 3% Ag-CaPp and (**d**) 5% Ag-CaPp nanoparticles in comparison to their control.

In addition, scratch assay presented in Figs. 5, 6 studied the effect of the as-prepared Ag-loaded CaPp nanoparticles samples on HSF cell migration. In the results of the wound-healing tests, the initial average diameter of the scratch area was about 3 mm and it was then measured after 0, 24, 48, and 72 h. After 24 h, the wound width was found to be 1.34 ± 0.062, 1.61 ± 0.024, 0.90 ± 0.081, 1.18 ± 0.24, 1.35 ± 0.44 for control, CaPp, 1.5% Ag-CaPp, 3% Ag-CaPp and 5% Ag-CaPp samples respectively while after 48 h, the wound width was found to be 0.05 ± 0.043, 0.28 ± 0.06, 0.00 ± 0.03, 0.35 ± 0.09 and 0.20 ± 0.03 for control, CaPp, 1.5% Ag-CaPp, 3% Ag-CaPp and 5% Ag-CaPp samples respectively. All samples showed complete wound closure (wound width = 0.00 mm) after 72 h. The difference in the wound closure width of some samples is related to the different initial wound width which was higher for high silver containing samples. From the results, we can deduce that the increase of silver ions content results in an increase in migration rate, as silver was reported to enhance wound healing and cell migration in many studies^56–59^. These results agree with results obtained by Moniri et al. in their study on nanocellulose/silver nancomposites as they found that the silver containing composite have higher migration rate than control cells because of the increased the expression level of different genes related to wound healing activity (*TGF*-β*1*, *MMP2*, *MMP9*, *CTNNB1*, and *Wnt4*) which indicates that silver increase wound healing activity^59^. The 1.5% Ag.CaPp sample showed the best migration rate compared to the control and CaPp samples and the rate begin to decline with higher concentrations which could be attributed to the disturbance of cell growth at higher concentration of silver ions as indicated from cell viability test. These results indicate that the loading of silver ions into the CaPp nanoparticles at low amount can better promote the invasion and migration of fibroblast cells, and enhance their synergistic effects.

### DNA Fragmentation assay

**Figure 7:**
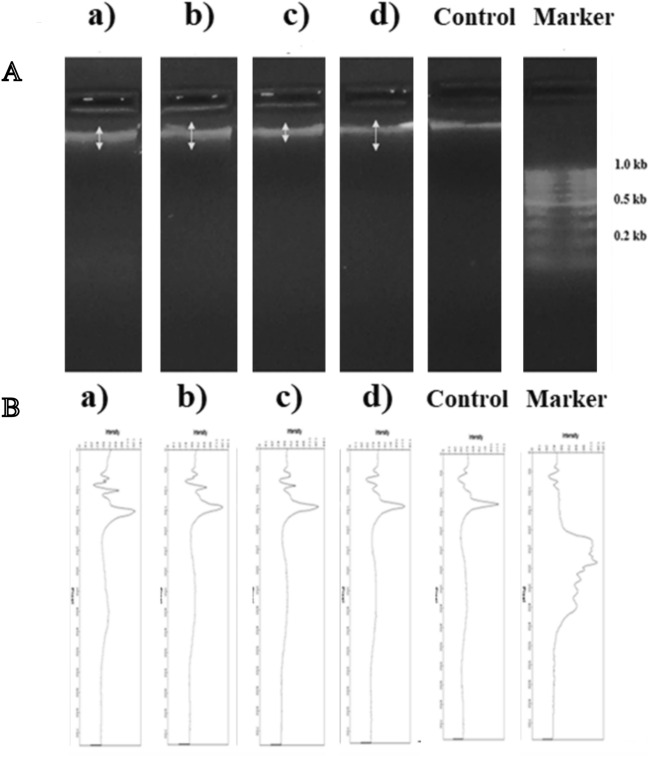
Detection of DNA fragmentation by (**A**) agarose gel electrophoresis. PC12 cells were exposed to different chemicals for 48 h, and then cells, were collected. Fragmented DNA was extracted from cells and electrophoresed on 1% agarose gels, stained with ethidium bromide, and photographed. (**B**) the intensity and fragmentation using a gel analyzer program of the samples: Control (untreated cells), Marker (DNA molecular weight marker), (a) CaPp, (b) 1.5% Ag- CAPp, (c) 3% Ag-CaPp and (d) 5% Ag-CaPp nanoparticles.

DNA gel electrophoresis was used to investigate whether the prepared nanoparticles can cause DNA fragmentation in PC12 cells. Cells were treated with various chemical compounds for 48 h, and then DNA was isolated from each treated sample. DNA fragmentation was assessed by gel electrophoresis. As shown in Fig. 7, according to comparing with control (not treated), CaPp, 1.5% Ag- CAPp, 3% Ag-CaPp and 5% Ag-CaPp did not induce any fragmentation with DNA concentrations. Also, DNA gel electrophoresis pattern was confirmed by determining the intensity and fragmentation using a gel analyzer program. Also, the data in Fig. 7a,b derived from the DNA fragmentation pattern were confirmed by Gel Analyzer analysis observed in gel electrophoresis the samples did not induce any fragmentation. The original DNA fragmentation is attached as a supplementary information.

## Conclusions

In this study three different concentrations of silver doped calcium polyphosphate were successfully prepared via wet chemical precipitation method. By increasing the silver percentage, the particle size and also the thermal stability increases. The samples showed good antibacterial properties against *E. coli, Staphylococcus aureus,* and *Enterococcus faecium.* The cytotoxic test showed that all the prepared samples were cytocompatible. The 3% Ag-CaPp showed the optimum cell migration rate and wound healing ability. DNA fragmentation test was done and all samples did not induce DNA damage. So, we can conclude that the prepared nanoparticles especially the 3% Ag-CaPp concentration would be a very promising material for bone and wound healing applications (Supplementary Information).

### Supplementary Information

Supplementary Information.

## Data Availability

The datasets used and/or analyzed during the current study are available from the corresponding author on reasonable request.
