# Supplementary figures and images for: Cell migration, DNA fragmentation and antibacterial properties of novel silver doped calcium polyphosphate nanoparticles
Source: Sci Rep. 2024 Jan 4;14:565. doi: 10.1038/s41598-023-50849-z (PMC10766647; doi:10.1038/s41598-023-50849-z)

Full length gel, 2 samples are not included in our study

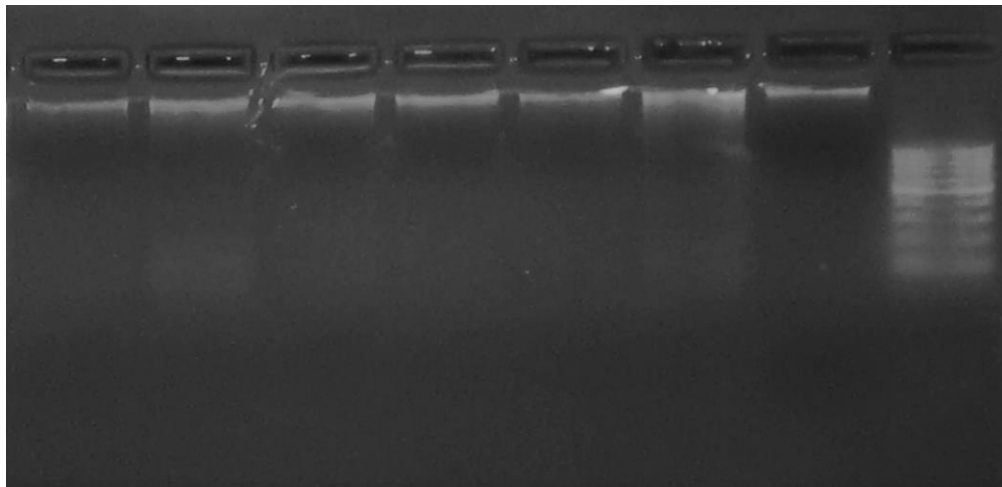

Supplement: Supplementary file 1 — Supplementary Information. [file 41598_2023_50849_MOESM1_ESM.pdf]
